# Endozoochory by mallard in New Zealand: what seeds are dispersed and how far?

**DOI:** 10.7717/peerj.4811

**Published:** 2018-05-23

**Authors:** Riley D. Bartel, Jennifer L. Sheppard, Ádám Lovas-Kiss, Andy J. Green

**Affiliations:** 1 Clayton H. Riddell Faculty of Environment, Earth, and Resources, University of Manitoba, Winnipeg, Canada; 2 School of Biological Sciences, University of Auckland, Auckland, New Zealand; 3 Department of Botany, University of Debrecen, Debrecen, Hungary; 4 Department of Wetland Ecology, Estación Biológica de Doñana, EBD-CSIC, Seville, Spain

**Keywords:** *Anas platyrhynchos*, New Zealand, Seed dispersal, Daily movement, Mallard, *Solanum nigrum*, Endozoochory, Polygonaceae

## Abstract

In Europe and North America waterfowl are major dispersers of aquatic and terrestrial plants, but in New Zealand their role has yet to be investigated. Mallards were introduced to New Zealand in the late 1800s, and today they are the most abundant and widespread waterfowl in the country. To assess seed dispersal, we radiomarked 284 female mallards from two study sites during the pre-breeding (June–August) and breeding (August–December) periods in 2014–2015, and examined movements that occurred within 24, 48 or 72 h when seed dispersal by endozoochory is considered likely. During June and July 2015, we collected 29 faecal samples from individual female mallards during radiomarking and 24 samples from mallard flocks. We recovered 69 intact seeds from the faecal samples and identified 12 plant taxa. Of the plant seeds identified and dispersed by mallards in this study, 40% were members of the Asteraceae family, nine plant species were alien to New Zealand, and the indigenous-status of three unidentified taxa could not be determined. Two taxa (and 9% of seeds) were germinated following gut passage: an unidentified Asteraceae and *Solanum nigrum*. During the pre-breeding and breeding periods, movement of females within 24 h averaged 394 m (SD = 706 m) and 222 m (SD = 605 m) respectively, with maximum distances of 3,970 m and 8,028 m. Maxima extended to 19,230 m within 48 h. Most plant species recorded are generally assumed to be self-dispersed or dispersed by water; mechanisms that provide a much lower maximum dispersal distance than mallards. The ability of mallards to disperse viable seeds up to 19 km within 48 h suggests they have an important and previously overlooked role as vectors for a variety of wetland or grassland plant species in New Zealand.

## Introduction

Endozoochory, the dispersal of propagules (e.g., seeds) through the gut by an animal, is a common dispersal strategy of many plant species (Reynolds & Cumming, 2016; Janzen, 1984; Wenny et al., 2016). In New Zealand, endozoochory is considered the second most frequent dispersal mechanism of plants and is reportedly used by 33% of native flora (Thorsen, Dickinson & Seddon, 2009). However, only 12% of native plant species produce fleshy fruits, and endozoochory of other plants depends mainly on granivory (Thorsen, Dickinson & Seddon, 2009). Granivory is often an over-looked pathway of seed dispersal (Costa et al., 2014; Green et al., 2016; Farmer et al., 2017), and although its significance has been recognized in New Zealand, no previous studies have documented seed dispersal by granivorous waterfowl there (Thorsen, Dickinson & Seddon, 2009).

Plants benefit from endozoochory because it allows them to colonise new sites, evade predators, and may provide a higher likelihood of germination (Wenny et al., 2016; Green et al., 2016). Often, plants coevolve with their dispersers, and plant populations may decline if their dispersers become endangered or extinct (Thorsen, Dickinson & Seddon, 2009). However, nowadays alien disperser species, which had no time to coevolve with the native plants, are widespread, particularly in New Zealand (Turbelin, Malamud & Francis, 2017). Some introduced animals can disperse seeds of native flora effectively and compensate for reductions in native vectors (Staddon, Compton & Portch, 2010). For instance, introduced birds in Hawaii have replaced the role of extinct frugivorous birds as the primary vectors of some native plants (Cox, 1983; Foster & Robinson, 2007). In New Zealand, introduced blackbirds (*Turdus merula*) and song thrushes (*T. philomelos*) dispersed more seeds than native frugivorous avifauna within secondary forested areas (Wyman & Kelly, 2017; but see Macfarlane, Kelly & Briskie, 2016). However, alien plants can also disperse by endozoochory and this could have negative consequences for native biodiversity and ecosystem functions (Reynolds, Miranda & Cumming, 2015; Green, 2016; Van Leeuwen, 2018).

In New Zealand, studies of avian endozoochory have largely focused on frugivorous avifauna in forested and native habitats (Wyman & Kelly, 2017; Clout & Hay, 1989). Nearly 60% of New Zealand is comprised of low-lying agricultural and pastoral land (MacLeod et al., 2008), which contains remnant patches of native forests, wetlands, rivers, and swamp areas, with a vast network of drainage ditches and ponds (e.g., Miskell, 1993; Buckland, 2008). While these modified habitats have become largely dominated by invasive plants (e.g., *Salix cinerea*, *Juncus bufonius*, *Rubus fruticosus, Ulex europeaus*), numerous native plant species such as raupō (*Typha orientalis*), harakeke (*Phormium tenax*), swamp kiokio (*Blechnum novae-zelandiae)*, manuka (*Leptospermum scoparium*) and toetoe (*Austroderia toetoe*) still inhabit remaining peatlands and swamps. However, little research has considered seed dispersal in these ecosystems and the role avian vectors may have on the maintenance of indigenous flora, or the expansion of invasive species, in such highly fragmented landscapes. Mallards (*Anas platyrhynchos*) are one of the most abundant birds in these habitats.

Waterbirds such as ducks, rails, and shorebirds are major vectors of dispersal for aquatic and terrestrial plants by endozoochory (Green et al., 2008; Green et al., 2016; Lovas-Kiss et al., 2018b). However, many of the extant native waterbird species such as brown teal (*Anas chlorotis*), South Island takahe (*Porphyrio hochstetteri*), Australasian bittern (*Botaurus poiciloptilus)*, and black stilt (*Himantopus novaezelandiae*) are endangered or threatened (Robertson et al., 2017), thus their role as vectors in New Zealand may be limited. Conversely, mallards were introduced to New Zealand in the late 1800s, and they became widespread following the combined release of ∼25,000 individuals during 1940–1960 and the genetic introgression between mallard and the native grey duck (Williams & Basse, 2006; Dyer & Williams, 2010; Guay et al., 2014). Today, mallards and their hybrids (hereafter mallards) are the most common and widespread waterfowl (Anatidae) species in the country, and although total population size is unknown, around 500,000 are hunted each year (Mcdougall & Amundson, 2017). Mallards use agriculturally dominated habitats and are major vectors of plant dispersal in Europe and North America (Soons et al., 2016; Green et al., 2013; Farmer et al., 2017; Lovas-Kiss et al., 2018b). During daily movements of mallards between foraging and roosting sites in autumn and winter (outside migration periods) in the Netherlands, 41% of egested seeds were dispersed away from the sites where they were ingested, over distances of up to 25 km away (Kleyheeg et al., 2017a). Given their abundance and widespread distribution, understanding endozoochory by mallards in New Zealand is important for conservation agencies who manage native and alien vegetation in areas where mallards reside.

Here, we evaluated seed dispersal of wild mallards and mallard-grey duck hybrids (*A. platyrhynchos* × *superciliosa*) in New Zealand. We combined mallards and their hybrids in our study because they exhibit similar behaviour and female mallards were difficult to distinguish from mallard-grey duck hybrids without the aid of genetic analysis. Specifically, we aimed to (i) identify plant species dispersed by mallards, (ii) determine the indigenous status and germinability of seeds egested by mallards, (iii) assess whether these plant species are recognized as having the capacity to disperse via birds, (iv) evaluate the likely dispersal distances for seeds by endozoochory, as determined by radio-tracking of mallards.

## Methods

### Study areas

During 2014–2015, we captured 243 pre-breeding mallards throughout two study areas in New Zealand (Fig. 1 in Sheppard et al., 2017). One site was located on the South Island, approximately 30 km north of Invercargill in the Southland District of the Southland Region (SOU) and another on the North Island, approximately 20 km south of Hamilton in the Waipa District of the Waikato Region (WAI). The Southland District was primarily comprised of ryegrass (*Lolium perenne*), cocksfoot (*Dactylis glomerata*) and clover (*Trifolium repens*) pastures for livestock (i.e., sheep, deer and dairy cattle) grazing. Swamp and peatland wetlands were once abundant in the Southland District, but have been converted to agriculture and the riparian margins of waterbodies are presently dominated by cutty grass (*Carex coriacea*), swamp sedge (*C. virgate*)*,* purei (*C. secta*)*,* Edgars rush (*Juncus edgariae*), buttercup (*Ranunculus repens*), and exotic grasses (Walls & Rance, 2003). However remnant habitat patches consist of kahikatea (*Dacrycarpus dacrydioides*) and matai trees (*Prumnopitys taxifolia*) as well as toetoe (*Austroderia fulvida*), flax (*Astelia fragrans*), hawthorn (*Crataegus monogyna*) and gorse (*Ulex europaeus*) that occur in drier, upland areas (Walls & Rance, 2003). The Waipa District predominantly consisted of dairy pastures within the catchment area of peat-lakes and peaty lowlands, including Lakes Ngāroto, Maratoto, Ruatuna, Rotomanuka and Mangakaware, which were bordered by grey willow (*Salix cinerea*), swamp cypress (*Taxodium distichum*), *Typha orientalis*, blackberry (*Rubus fruticosus*), gorse, mercer grass (*Paspalum distichum*), sharp-spiked sedge (*Eleocharis acuta*) and *Carex secta* (Champion, De Winton & De Lange, 1993; Thompson & Greenwood, 1997). However, reclamation and replanting of indigenous wetland plants have been ongoing at some lakes for several years.

### Monitoring procedures

Each year, we captured mallards using baited funnel traps (Bub, 1991) placed on the edge of refuge ponds (i.e., ponds that were not hunted during the May hunting season). We baited four trap sites with barley (*Hordeum vulgare*) in Southland and six trap sites with feed corn (*Zea mays*) in Waikato. We baited sites from six weeks prior to trapping through to completion of trapping (SOU_2014_: 5 July–17 July; SOU_2015_: 3 July–15 July; WAI_2014_: 4 June–22 June; WAI_2015_: 2 June–17 June) during which we re-baited the traps every 1–3 days. Once trapping commenced, we closed the traps each morning and transferred females to communal holding pens to await processing and surgical implantation of a 22 g intra-abdominal radio-transmitter as part of an associated study (Sheppard, 2017; Sheppard et al., 2017). We layered holding pens with a sheet of plastic to collect faecal samples (2015 only) and prevent the contamination from foreign material already on the ground. Once females were equipped with the transmitter, we placed them in solitary recovery pens for at least 45 min then released them back into the wild (see Sheppard et al., 2017 for details). In both years, trapping continued until ∼60 females per site were marked (5–19 days).

The day following transmitter deployment, we began radio-tracking marked females every 1–3 days using hand-held telemetry (homing) or triangulated locations using truck-mounted, null-array, antenna systems (Kenward, 1987) and Location of a Signal Software, version 1.03 (LOAS; Ecological Software Solutions, Hegymagas, Hungary). Females were approached on foot weekly or whenever investigators triangulated birds to the same location more than once. When a female was located, we recorded the date, time, type of location (homing or triangulation), and geographic coordinate of the female. If females began nesting, nests were subsequently checked every 7–10 days until nest fate was determined. Following hatch, we tracked females with broods every 1–5 days. Otherwise, we tracked females weekly to monitor renesting attempts by approaching them on foot.

From late August to early November, we searched roadsides, riparian edges of drainage ditches, lakes, ponds, and other dense-nest habitats throughout the study area to find nests of unmarked mallards and, when possible, trapped nesting females (*n* = 61) during late incubation and equipped them with 9 g back-mounted prong-and-suture radio-transmitters (Model LB-66, telonics, Mesa, Arizona; Rotella et al., 1993; Paquette et al., 1997). These females were tracked using the above protocol (Sheppard, 2017). All marking and tracking procedures were approved under University of Auckland Animal Use Permit 001331 and Department of Conservation Field Permit 38732-FAU.

### Faecal sample collection, preparation, and processing

In 2015, we collected faecal samples opportunistically from females during trapping and handling, and from flocks of mallards in the field while conducting radio-telemetry (Table 1). To avoid contamination, the portion of the faecal sample that was in contact with the ground was scraped away using stainless steel metal. If analysing samples the same day as collection, we placed faecal samples in sealed plastic containers and stored them in a fridge at 1.6 °C (*n* = 22). Otherwise, we placed samples in envelopes and dried them at room temperature (*n* = 31). Prior to analysing dried samples, we re-hydrated the sample by soaking it in non-chlorinated water. During analysis, we used a digital scale to measure the fresh weight of each sample (±1 g). We used non-chlorinated water and two stacked sieves (63 and 250 µm) to separate digested material and filter out intact, whole seeds, which we identified based on morphology (Cappers, Bekker & Jans, 2012; James et al., 2012; Webb & Simpson, 2001). In our results, we present full data on seeds which had an intact seed coat only. However, at least a small proportion of any seed type present in the diet usually survives gut passage (Soons et al., 2016). We therefore identified one other taxon for which only broken seeds were recorded, as evidence for endozoochory.

**Table 1:** Details of mallard faecal samples. The location, number, and type (individual or flock) of faecal samples collected from mallards during marking or radio-tracking in two study sites in New Zealand during June–July, 2015.

Collection site	Latitude (′S)	Longitude (′E)	Individual^a^	Flock^b^
Southland				
Calder Road	46° 13.079	168° 18.512	–	11
Forbes Road	46° 13.924	168° 19.241	–	4
Kean Road	46° 11.840	168° 17.867	6	–
Lochiel	46° 13.095	168° 19.670	7	–
Thompson Crossing	46° 11.446	168° 17.955	3	–
Waikato				
Forkert Road	37° 55.219	175° 15.754	1	2
Lake Rotoroa	37° 47.884	175° 16.193	1	–
McGregor Road	37° 53.323	175° 15.442	1	–
Ngahinapouri	37° 54.524	175° 14.087	–	1
Ohaupo	37° 54.206	175° 18.333	2	–
Paterangi	37° 57.343	175° 14.670	8	6

**Notes.**

a Sample collected from the recovery pen or bag used to weigh an individual female during marking and processing of transmitter.

b Sample collected from the bait trap or holding pen where flocks of ducks were held during marking, or from an area where a flock of ducks flushed during radio-tracking.

We shipped seeds from New Zealand to Spain for identification and processing. We determined whether seeds were viable by testing all intact seeds for germinability, using tissue plates filled with moistened filter paper within a germination chamber. We set the chambers at 12 h of light at 24 °C and 12 h of darkness at 18 °C, and ran the germinability tests for two months. For plant species present in the European flora, we obtained the dispersal syndrome from Julve (1998). These syndromes are identified based on an inspection of the morphology of the diaspore, and are generally assumed to reflect the dominant dispersal mechanism for a given taxon.

### Movement analysis and seed dispersal distance

Unlike Kleyheeg et al. (2017a) and Kleyheeg et al. (2017b), we did not have data on continuous locations that would allow us to predict seed dispersal distance with a high resolution. However, mallards make predictable daily movements between roosting and foraging sites (Kleyheeg et al., 2017b; Bengtsson et al., 2014), so we expected that our long (typically daily) intervals between radio-locations would pick up these movements on some occasions. Our dataset is conservative, since mallards will often likely have moved between locations but returned to the same site before the subsequent tracking event (i.e., the movement was often missed). To compensate, we limited our analysis to tracking events that occurred subsequently within 72 h because although most intact seeds are egested within 12 h of ingestion, a small proportion are egested in a viable state between 24–72 h after ingestion (Garcia-Alvarez et al., 2015; Farmer et al., 2017). Hence, our tracking dataset is expected to underestimate the mean or median dispersal distance for seeds, but is not likely to overestimate the maximum dispersal distance.

We calculated the number of hours between bird movements and determined the distance moved between each consecutive movement using the ‘adehabitat’ package (Calenge, 2006) in R*3.3.0 (R Core Team, 2015). We excluded any movements that occurred beyond our 72 h limit and classified movements into one of three categories: (i) within 24 h of the last tracking events (<24 h); (ii) between 24 to <48 h of the last tracking event; and, (iii) between 48–72 h. Some birds went missing (were not located within 10 km of the study area), died, or their transmitters fell off, thus tracking records varied for each female and ranged from one day to six months (end of breeding season).

## Results

### Endozoochory

In 2015, we collected 53 faecal samples including 29 samples from individual female mallards during capture and 24 samples from flocks during radio-tracking (Table 1). The number of seeds (intact and broken) per sample ranged from 0–27 ($\bar {x}=3.21$, SD = 5.03) and average mass of sizable samples (≥1 g) was 5.97 g (SD = 11.18, range = 1–52 g; *n* = 29). Of the 170 seeds recovered, 69 were intact, from which we identified 12 taxa (Table 2). Two of these were identified to family level, one to genus level, and nine to species level (Table 2). We also recorded broken seeds of *Setaria verticillata* (Poaceae). Aside from unidentified Asteraceae and *Ranunculus repens* that occurred in both sites, plant taxa differed between study areas (Table 2).

**Table 2:** Plant taxa dispersed by endozoochory. Plant taxa collected from faeces of mallard ducks in New Zealand, 2015 from two sites (SOU, Southland; WAI, Waikato), the number of samples that had whole/intact seeds (WS), the total number of intact seeds collected per species (TS), and the number of seeds which germinated (*N* germ).

Family	Taxa	Vernacular and Māori Name	WS^a^	TS^b^	*N* germ	Site	Dispersal syndrome^c^
Amaranthaceae	Chenopodium album^*^	Fathen/ Hua inanga	1	1	0	WAI	Barochory
Asteraceae	Undetermined	–	5	27	1	Both	–
Boraginaceae	*Myosotis* sp.	Forget-me-not	1	1	0	SOU	–
Cyperaceae	Undetermined	–	1	1	0	WAI	–
Poaceae	Agrostis stolonifera^*^	Creeping bent	1	1	0	SOU	Barochory
	*Hordeum vulgare*^d^^*^	Barley	5	6	0	SOU	Epizoochory
	Paspalum dilatum^*^	Paspalum	3	3	0	WAI	Barochory
Polygonaceae	Polygonum aviculare^*^	Wireweed/ Makahakaha	2	2	0	SOU	Barochory
	Persicaria hydropiper^*^	Water pepper	2	3	0	WAI	Hydrochory
	Persicaria maculosa^*^	Willow weed	6	11	0	SOU	Barochory
Ranunculaceae	Ranunculus repens^*^	Creeping buttercup	3	4	0	Both	Epizoochory
Solanaceae	Solanum nigrum^*^	Black nightshade/Raupeti	1	9	5	WAI	Endozoochory

**Notes.**

a The number of samples that had intact seeds of each species.

b Total number of seeds collected from all samples.

c Morphological syndrome obtained from Julve (1998).

d Taxon was likely ingested as bait during trapping.

* Alien species to New Zealand.

All nine species identified are alien to New Zealand but listed in the European flora (Table 2). Of these, five species are reported to be self-dispersed (barochory), one to disperse via water (hydrochory) and two to disperse by epizoochory (transportation on external surfaces of an animal, see Coughlan et al., 2017). Only *Solanum nigrum* has an endozoochory syndrome (Table 2). Overall, the unidentified Asteraceae was the most abundant taxon recorded (27 seeds; Table 2), but it remains unclear whether this is a native or invasive species. The second most abundant taxon was the alien *Persicaria maculosa* (*n* = 11 seeds; Table 2). Only 9% (*n* = 6) of intact seeds germinated within our two month trial; one was unidentified Asteraceae and five were *Solanum nigrum* (which represented 55% of the seeds collected and tested for this taxon).

### Mallard movements

Within the 72 h seed dispersal period, we collected 3,887 consecutive observations (homing = 3,863, triangulation = 130) including 2,059 records during pre-breeding and 1828 records during breeding (including laying, incubation, renesting intervals, and brood-rearing). Mean error ellipse polygon of triangulations was 0.75 ha (SD = 1.50 ha; range = 0.0001–8.88 ha). We summarised pre-breeding and breeding periods separately because activities during breeding are typically constrained to a nesting site or breeding territory.

**Figure 1:**
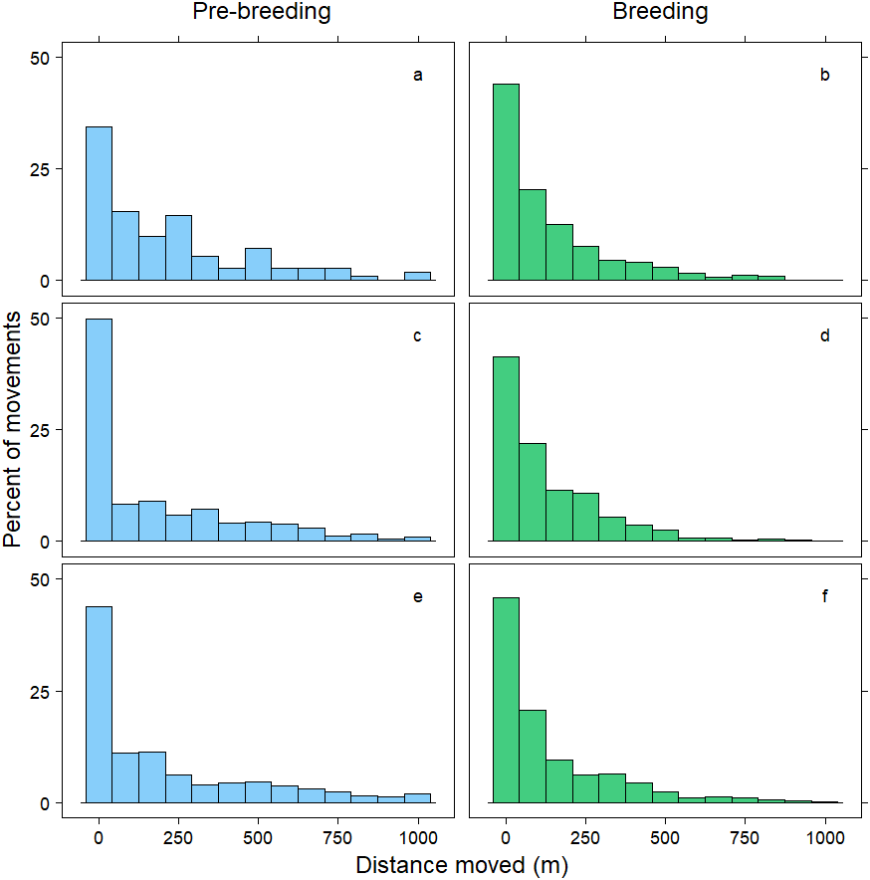
Distribution of movements up to 1,000 m. Distance moved (m) by female mallards within 24 h (A, B), between 24–<48 h (C, D) and between 48–72 h (E, F) during the pre-breeding (blue) and breeding (green) periods. Only movements less than 1,000 m are illustrated; representing 90% of the data.

**Figure 2:**
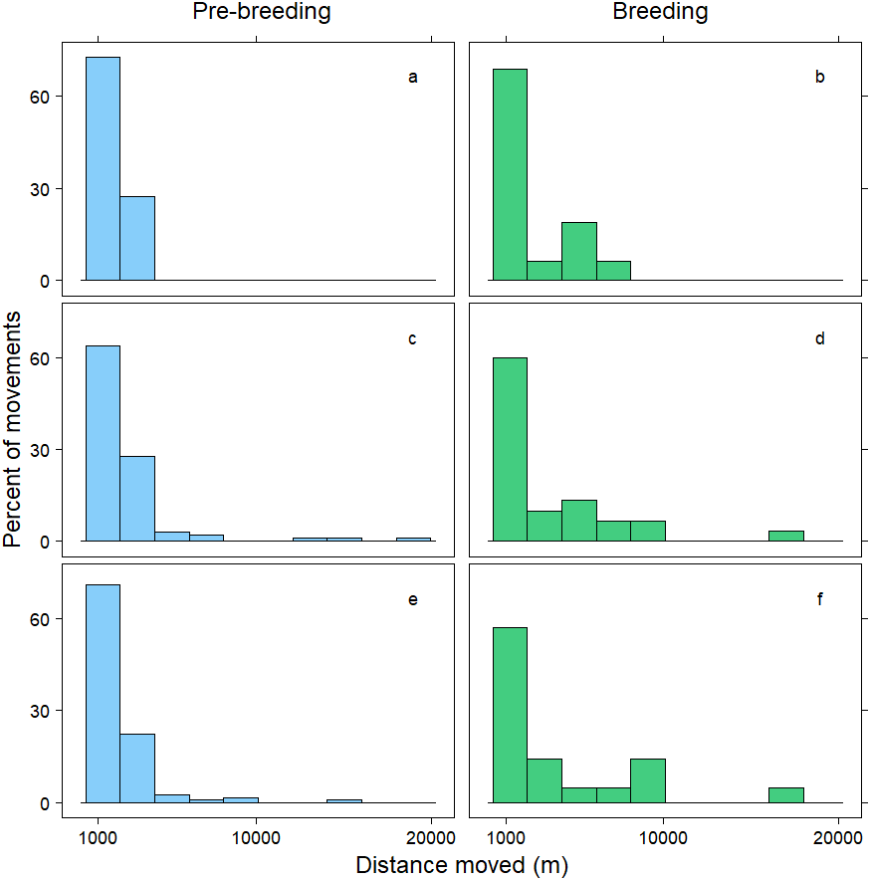
Distribution of movements over 1,000 m. Distance moved (m) by female mallards within 24 h (A, B), between 24–<48 h (C, D) and between 48–72 h (E, F) during the pre-breeding (blue) and breeding (green) periods. Only movements greater than 1,000 m are illustrated; representing 10% of the data.

Within 72 h, the mean distance travelled between consecutive observations was 392 m (SD = 1,066 m), but birds moved an average of 258 m further during pre-breeding than during the breeding period (*t* =  − 7.70, *p* < 0.001; Table 3). Approximately 90% of relocations occurred within 1,000 m of the previous tracking location, and during both the pre-breeding and breeding periods most of these movements were less than 200 m (Fig. 1). The fraction of movements exceeding 500 m was greater during pre-breeding, but there were no obvious differences in the distances moved between the <24 h, 24–<48 h and 48–72 h intervals (Fig. 1). However, 10% of movements exceeded 1000 m, and all the movements recorded over 10 km occurred between 24–72 h (Fig. 2). Thus, although no movement was reported in 30% of consecutive observations (i.e., the bird was in the same location during the previous tracking event), the maximum distance between tracking events was 8,028 m within 24 h, 19,230 m within 24–48 h, and 16,640 m within 48–72 h (Fig. 2; Table 3).

**Table 3:** Distance moved by mallards. Sample size (*n*), mean ($\bar {x}$), standard deviation (sd), and maximum (max) distance (m) travelled within 24 h, between 24–48 h, and between 48–72 h during the pre-breeding and breeding periods of female mallards equipped with radio-transmitters in Southland and Waikato, New Zealand, 2014–2015.

	<24 h				Between 24–48 h				Between 48–72 h			
	*n*	$\bar {x}$	sd	max (m)	*n*	$\bar {x}$	sd	max (m)	*n*	$\bar {x}$	sd	max (m)
Pre-breeding	122	393.7	705.6	3,969.6	876	451.7	1,206.4	19,230.7	1,061	578.3	1,177.1	16,017.2
Breeding	461	221.8	605.8	8,028.1.4	842	255.1	932.2	16,643.9	523	284.8	1,104.7	16,640.1

## Discussion

To our knowledge, this was the first study to investigate dispersal of seeds in New Zealand by waterfowl. Previous studies in the southern hemisphere have shown that seed dispersal by granivorous waterfowl is an important process, but did not include tracking the movements of the vectors (Green et al., 2008; Raulings et al., 2011; Reynolds & Cumming, 2016). In a large sedentary population of mallards and mallard-grey duck hybrids, we found that females dispersed intact seeds of at least 12 different plant taxa. These included nine alien species and three unidentified taxa which may have been of native origin. Many of the alien species we identified have been reported throughout New Zealand, but there have been few records of *Agrostis stolonifera*, *Polygonum aviculare*, and *Persicaria maculosa* occurring in the Southland region, which suggests that these species have recently extended their range southwards (AVH, 2017).

**Figure 3:**
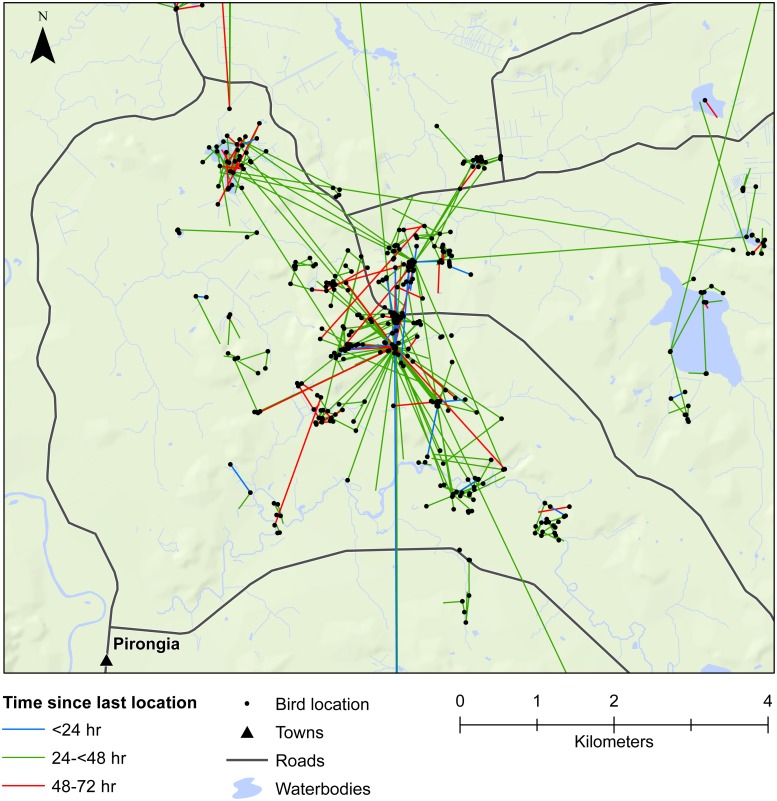
Movements within the Waikato study area. Movement of female mallards within the Waikato study area between consecutive relocation events within 24 h (blue line), 24–<48 h (green line), or 48–72 h (red line).

**Figure 4:**
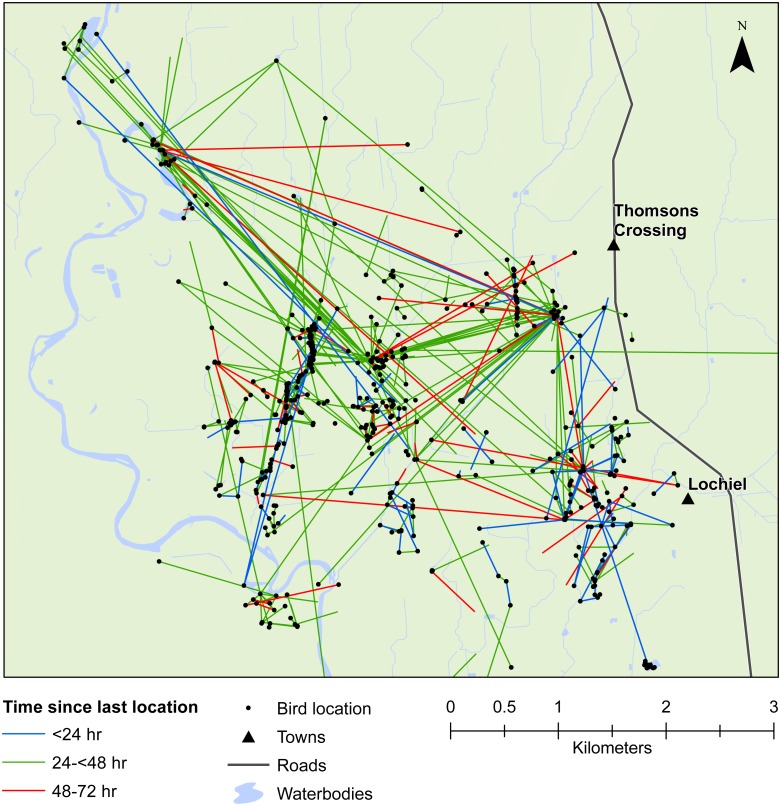
Movements within the Southland study area. Movement of female mallards within the Southland study area between consecutive relocation events within 24 h (blue line), 24–<48 h (green line), or 48–72 h (red line).

We also demonstrated that female mallards were capable of moving up to 8 km within 24 h, or up to 19 km within 48 h. Our telemetry data indicated that mallards frequently moved between various waterbodies or between pastoral land and wetlands (Figs. 3 and 4). Thus, there is high potential for mallard-mediated seed dispersal for a range of wetland or pastoral species in New Zealand, including native and alien flora. Given the abundance of mallards throughout the country (Robertson, 2007), they are clearly important vectors of plants, as has previously been demonstrated for mallards in agricultural landscapes in Europe and North America (Soons et al., 2016; Farmer et al., 2017; Kleyheeg et al., 2017a).

Our results also provided important insights into seed dispersal mechanisms. Of nine plant species identified, endozoochory was recognised as the dispersal mechanism only for *Solanum nigrum* (Table 2). Most of the other species are generally assumed to disperse via barochory (self-dispersal) or hydrochory, yet these dispersal mechanisms provide much shorter dispersal distances than those expected from endozoochory by mallards (Bullock et al., 2017). Of particular importance are the high maximum dispersal distances (MDDs) of up to 19 km provided by mallards, since MDD has a vital influence on plant migration and colonization rates, including rates of spread of alien species (Bullock et al., 2017). In highly fragmented areas, such as the agriculturally dominated regions of Southland and Waikato, plants which disperse via endozoochory are better able to exploit patchy and isolated habitats (Soons et al., 2016; Kleyheeg et al., 2017a). As such, waterbird-mediated dispersal of indigenous flora may be especially important for the conservation of threatened or endangered plants, whereas endozoochory of alien species may aid in their expansion (Green, 2016). Even for plants able to disperse via water, mallards are vital for dispersal between catchment areas (Figs. 3 and 4). Many of the alien plant species we identified are already ubiquitous throughout New Zealand, and waterfowl may have played an important historical role in their spread. *Persicaria hydropiper* and *S. nigrum* are both widespread throughout the country (Champion & Hofstra, 2013; AVH, 2017), and the other species we identified appear to have spread beyond their previously reported distributions.

Given the limited sample size and timing of our study, we probably only detected a small proportion of the plant taxa that mallards disperse in New Zealand. Sample size effects detected in other endozoochory studies on mallards elsewhere indicate that the number of plant taxa detected increases with sample size (Soons et al., 2016; Lovas-Kiss et al., 2018b). We collected faecal samples in June and July during the late Austral-winter when many indigenous plant species are dormant but invasive species are fruiting (Wotton & McAlpine, 2015). Hence, the availability and abundance of diaspores from native flora may have been limited during our collection period. Whether mallards play an active role in dispersing endemic plants during peak floral-fruiting in Austral-spring to autumn should be investigated.

We confirmed the germinability of two plant taxa following gut passage. Many species did not germinate during our tests, perhaps because the protocols we used were not ideal. The long delay between collection and germination trials in different continents and the changes in humidity and temperature of seeds during this period likely reduced germinability rates. Previous studies of mallards have shown that intact seeds tend to maintain higher germinability after gut passage (Garcia-Alvarez et al., 2015; Van Leeuwen et al., 2012; Farmer et al., 2017), so we suspect that under better conditions more seeds may have germinated successfully. Indeed, viability after gut passage has been demonstrated for some of these same species during previous studies of mallards elsewhere (Farmer et al., 2017). Conversely, approximately 55% of *Solanum nigrum* seeds germinated. *S. nigrum* is a noxious, invasive weed common in cropping soils in New Zealand (Shah, Falloon & Bulman, 2010) and can remain dormant for extended periods of time (Edmons, 1983). Possibly, extended dormancy of *S. nigrum* enabled it to withstand the storage and transport from New Zealand to Spain, and may explain why the species was so successful in our trials.

The mean distance moved by female mallards between consecutive observations was 392 m (0.39 km), yet the longest distance recorded in this study was 8.0 km within 24 h and 19.2 km within 48 h. Given that seed dispersal is highly likely within 24 h of ingestion (Garcia-Alvarez et al., 2015; Kleyheeg et al., 2017a), our results suggest that mallards were most likely to disperse seeds within 8.0 km of the core home range, but that movements up to 20 km also occurred. Our irregular tracking meant that we underestimated the frequency of movements, yet our results are generally similar to Kleyheeg et al. (2017b) who used continuous tracking and reported that average and maximum flight distances of mallards in the Netherlands was 0.4 km and 25.5 km, respectively. Our results demonstrate that mallards allow dispersal of plants in New Zealand over greater distances than those predicted based on seed morphology and associated syndromes (see Bullock et al., 2017 for distances for other dispersal mechanisms). However, they also illustrate how endozoochory by mallards can result in much shorter dispersal distances than those predicted by studies assuming that they fly continuously in a straight line during the interval between seed ingestion and egestion (e.g., Raulings et al., 2011; Farmer et al., 2017).

We also found that, on average, mallards moved slightly further per day during the pre-breeding period (early June–early August) relative to the breeding period. Prior to breeding, birds are feeding in flocks and beginning to establish breeding territories, and most movements occurred between feeding and roosting areas. However, some of the longer movements occurred during the breeding period and were related to nesting, incubation recesses and brood-rearing. Breeding movements may be greatest during breeding-phase transition periods (i.e., between nesting and brood-rearing areas, or renesting attempts). Only females were radio-marked, and it is likely that males exhibited fewer constraints and showed greater movement during the breeding period, as only females incubate eggs and attend broods. The non-breeding period coincides with peak fruiting of flora that are indigenous to New Zealand, and movements of mallards or other waterbirds during this time period are likely to be more extensive and should be further evaluated.

## Conclusion

Since their initial introduction into New Zealand ∼150 years ago, mallards have become the dominant waterfowl, and now play an important role as vectors of seed dispersal by endozoochory as observed in their native range. Most of the plants dispersed in our study were aliens, including all nine taxa identified to species, such that an alien bird is dispersing alien plants. With one exception (*S. nigrum*), these are not plants that have a fleshy fruit and hence an “endozoochory syndrome”, but rather are dry achenes (Julve, 1998) that are attractive to granivorous birds such as ducks. In New Zealand, as elsewhere, seed morphology has been used to identify putative mechanisms of dispersal for the native flora (Thorsen, Dickinson & Seddon, 2009). Generally, fleshy fruits are diagnostic of endozoochory, and researchers often focus on frugivores as plant vectors rather than granivorous waterbirds (Green et al., 2016). Recent studies from Europe have demonstrated that a range of waterbirds act as regular vectors by endozoochory for a wide variety of plants assigned to different dispersal syndromes (Van Leeuwen et al., 2017; Lovas-Kiss et al., 2018b; Lovas-Kiss et al., 2018a). In the absence of empirical evidence based on data on dispersal in field situations, diaspore morphology should not be considered as a reliable indicator of dispersal mechanisms. Ours is only a preliminary study of seed dispersal by waterfowl in New Zealand, and future work is required to establish how important mallards and native waterbirds are for the conservation of native flora or the spread of alien species.

##  Supplemental Information

10.7717/peerj.4811/supp-1 Supplemental Information 1 The raw data used to evaluate movements of mallards within a 72 hr period when seed dispersal is likely Variable list: x, eating; y, northing; date, date and time of the observation; dist, distance travelled between consecutive observations (m); dt, time between consecutive observation (seconds); id, ID/band number of bird; Breed, stage of the annual cycle (pre-breeding or breeding); dt2, time between consecutive observation (hours); dist2, distance travelled between consecutive observations (km); mvmt, period of seed dispersal ( 1 =  < 24 hrs; 2 = 24– < 48 hrs; 3 = 48–72 hrs).

## Additional Information and Declarations

### Field Study Permissions

The following information was supplied relating to field study approvals (i.e., approving body and any reference numbers): All marking and tracking procedures were approved under University of Auckland Department of Conservation Field Permit 38732-FAU.

### Data Availability

The following information was supplied regarding data availability: The raw data are provided as a Supplemental File.
